# Epidemic spreading with activity-driven awareness diffusion on multiplex network

**DOI:** 10.1063/1.4947420

**Published:** 2016-04-21

**Authors:** Quantong Guo, Yanjun Lei, Xin Jiang, Yifang Ma, Guanying Huo, Zhiming Zheng

**Affiliations:** 1 School of Mathematics and Systems Science, Beihang University, Beijing 100191, China; 2 Key Laboratory of Mathematics Informatics Behavioral Semantics (LMIB), Ministry of Education, China; 3 School of Mathematical Sciences, Peking University, Beijing 100191, China; 4 Center for Complex Network Research, Department of Physics, Northeastern University, Boston, Massachusetts 02115, USA

## Abstract

There has been growing interest in exploring the interplay between epidemic spreading with human response, since it is natural for people to take various measures when they become aware of epidemics. As a proper way to describe the multiple connections among people in reality, multiplex network, a set of nodes interacting through multiple sets of edges, has attracted much attention. In this paper, to explore the coupled dynamical processes, a multiplex network with two layers is built. Specifically, the information spreading layer is a time varying network generated by the activity driven model, while the contagion layer is a static network. We extend the microscopic Markov chain approach to derive the epidemic threshold of the model. Compared with extensive Monte Carlo simulations, the method shows high accuracy for the prediction of the epidemic threshold. Besides, taking different spreading models of awareness into consideration, we explored the interplay between epidemic spreading with awareness spreading. The results show that the awareness spreading can not only enhance the epidemic threshold but also reduce the prevalence of epidemics. When the spreading of awareness is defined as susceptible-infected-susceptible model, there exists a critical value where the dynamical process on the awareness layer can control the onset of epidemics; while if it is a threshold model, the epidemic threshold emerges an abrupt transition with the local awareness ratio *α* approximating 0.5. Moreover, we also find that temporal changes in the topology hinder the spread of awareness which directly affect the epidemic threshold, especially when the awareness layer is threshold model. Given that the threshold model is a widely used model for social contagion, this is an important and meaningful result. Our results could also lead to interesting future research about the different time-scales of structural changes in multiplex networks.

Network modeling plays an important role in identifying statistical regularities and structural principles common to many complex systems. As a representative way to describe the rapidly changing interactions among individuals, time varying network is gaining more and more interest. Hence, in order to explore the relationship between two processes accounting for the spread of epidemics, and the spread of awareness information to prevent its infection, we propose a multiplex network model with two layers, of which the information spreading layer is a time varying network and the contagion layer is a static network. By extending the microscopic Markov chain approach (MMCA), we are able to derive the epidemic threshold analytically. The numerical results show high accuracy compared with extensive Monte Carlo (MC) simulations, regardless of whether the information spreading process is defined as the susceptible-infected-susceptible model (SIS) model or the threshold model. Specifically, when the spreading of awareness is defined as the SIS model, there exists a critical value where the dynamical process on the awareness layer can control the onset of epidemics; while if it is a threshold model, the epidemic threshold emerges an abrupt transition with the local awareness ratio ***α*** approximating 0.5. The results also show that changes in the topology of the awareness layer could change the location of the meta-critical point, which is particularly important since a slight delay or advance of the epidemic could have important implications to vaccination strategies. In sum, our results give us a better understanding of the coupled dynamical processes and highlight the importance of taking the spread of information into consideration in the control of epidemics.

## I. INTRODUCTION

The problem of modeling how epidemics spread among individuals has been extensively studied by people from various fields for many years.^1–5^ In the field of physics, most approaches to these problems are related to the theory of phase transition, statistical physics, and critical phenomenon.^6–10^ Especially in last decades, the study of complex networks has provided new grounds to the understanding of epidemic dynamics for physicists.^11,12^ They proposed various models, such as the classical susceptible-infected-susceptible model (SIS),^13^ susceptible-infected-recovery model (SIR),^14^ and so on,^15,16^ to shed light on modeling the epidemic dynamics. Different models have addressed the problem from different perspectives, e.g., the frequency of contacts between people,^17^ duration of the disease,^18^ and risk perception.^19^

Furthermore, in network modeling of a single layer topology structure, the standard approach assumes that all links have the same activation time, which is clearly an abstraction of any real topology structure and cannot reveal the true relationships in the complex system very well.^20^ Therefore, as a natural way to describe the interrelated complex connections among individuals, the multiplex network is gaining more and more attention. It can overcome the drawbacks of the simplex network in which individuals interact only through one network, while in reality (same as in epidemic and information spreading), the same set of nodes might interact through multiple networks with different topologies and dynamical properties.^21–25^ Each layer could have particular features that are different from the rest, which make the multiplex network define a richer structure of interactions.^26^ Under the framework of the multiplex network, there has been growing interest in exploring the interplay between epidemic spreading with human response,^27–33^ since it is natural for people to take measures when they become aware of epidemics. Two interdependent layers can be used for modeling different dynamical processes, and the interactions between layers represent the coevolution of the processes. Several works have studied the problem by changing the model of information spreading, such as by considering the information spreading process as a SIS model; Granell *et al*.^27^ discovered the emergence of a metacritical point where the diffusion of awareness is able to control the onset of epidemics, whereas Guo *et al.*^28^ replaced the SIS model by a threshold model and found a two-stage effect on epidemic threshold. Besides, Granell *et al.*^29^ also analyzed the consequences of a massive broadcast of awareness (mass media) on the final outcome of the epidemic incidence, and the results showed that mass media could make the metacritical point disappear.

Until now, people pay much attention on the spreading model for the multiplex network, while the topology structure of it has gained little attention. In other words, all these models can be defined as connectivity model, where connections among individuals are long-lived elements.^34–37^ However, with the development of various kinds of social networks, in many cases, the interactions among individuals are rapidly changing and are characterized by processes with duration being defined on a very short time scale, such as information diffusion over an email, mobile calls among individuals, and so on.^38^ Since a time-aggregated representation of network's interactions neglects the time-varying nature of real systems,^35^ it is meaningful for us to model the information layer as a time varying network and to study the effects that information spreading process has on epidemic spreading. This scenario is common in reality if we consider the spreading of epidemic information originating from social media, for example, the spreading of Severe Acute Respiratory Syndromes. During this period, people can know about this epidemic from many sources, such as television (TV), radios, and Internet. Let us take TV as an example: the announcer can be regarded as an activated node and the connections between him and listeners are time-varying, which makes the proper topology of the connections be a time-varying network. At the same time, in order to decrease the infection probability, everyone needs to avoid contacts with strangers. And then, it is obvious that the contagion spreading layer can be treated as a static one. Therefore, in this paper, to fill this gap, a multiplex network with two layers is built. Specifically, the information spreading layer is a time varying network generated by the activity driven model,^34^ and the other one is a static network. We extend the microscopic Markov chain approach (MMCA)^27^ to derive the epidemic threshold of the model analytically. Compared with extensive Monte Carlo (MC) simulations, the method shows high accuracy for the prediction of the epidemic threshold. Besides, by considering different spreading models, namely, the SIS model and the threshold model, of the time varying network, we explored the interplay between epidemic spreading with information spreading. The results show that the coexistence of antagonistic spreading effects raises interesting physical phenomena, such as not only makes the epidemic threshold become larger but also lowers the density of the final epidemic size. Corresponding to different models, the metacritical point and two-stage effects also exist in the time varying cases, which verify again that these phenomena are rooted in the competition principle of the multiplex network model. Furthermore, we also find that the temporal changes in the topology hinder the spread of awareness which directly affect the epidemic threshold, especially when the awareness layer is the threshold model.

## II. MODEL DESCRIPTION

**FIG. 1.**
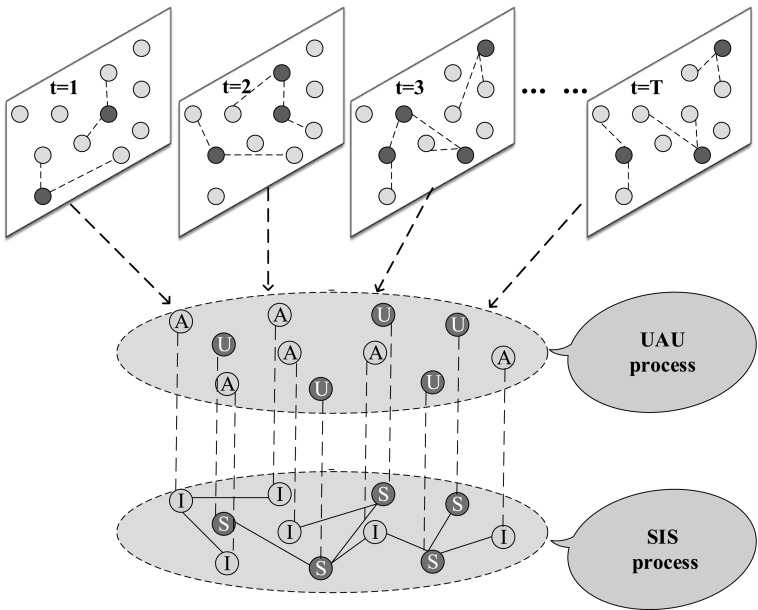
A simple example of the structure of the multiplex network proposed in the paper. The awareness layer is a time varying network where the spreading of awareness happens. At each time step, the topology structure of the awareness layer is built according to the activity driven model. Relying on the newly built network, the individuals interact with each other to change the states: unaware (green node) and aware (yellow node). The other layer corresponds to the network where epidemic transfers among two kinds of nodes, namely, susceptible (green node) and infected (red node). Individuals on two layers are the same. In particular, only three kinds of states exist in the multiplex network: unaware and susceptible, aware and infected, and aware and susceptible. Note that in the figure, red individuals on the time varying network represent the activated ones and each activated individual can have 2 connections with other individuals at each time step.

Let us start by defining the specific setup of the multiplex network we analyze. The awareness layer corresponds to the spread of information about epidemics, where individuals can be aware or unaware. Besides, there is a certain probability for the two states transforming between each other. More specifically, aware individuals have a probability of *δ* forgetting epidemics while unaware individuals can become aware with the probability *λ* through communication with aware individuals. As an important way to describe information diffusion process, the activity driven model is used to generate the connections among individuals on the awareness layer.^34^ Meanwhile, on the epidemic layer, there are two categories of individuals, i.e., S (susceptible) individuals and I (infected) individuals. Furthermore, the contagion layer, where the epidemic spreading process described by the classic SIS model occurs, is a static one. Because of the existence of coupled dynamical processes, it produces three kinds of states for each individual, including US (unaware and susceptible), AS (aware and susceptible), and AI (aware and infected), as illustrated in Fig. 1. Under the assumption of the SIS model for the spreading of epidemic, a susceptible individual can be infected by an infected neighbor with the probability *β*, and at the same time, an infected individual can recover to be susceptible with probability *μ*. If an individual is infected, it is natural that this individual becomes aware of the epidemic. However, if one susceptible individual is aware of the epidemic, the infectivity *β* can be reduced by a factor, which results from individual's behavioral responses to the presence of the epidemic.^27^ We use *β^U^* and *β^A^* to represent the infection rates without and with awareness, respectively. For the sake of simplicity, we will present the results for $\beta^{A}=0$ in this paper, which correspond to complete immunity for aware individuals.

## III. DYNAMICAL MMCA METHOD FOR OUR MODEL

In order to analyze the coupled dynamical processes on top of the multiplex network, we need to explore the details of the model. As described above, the multiplex network consists of two layers with different topology structures. The awareness layer is a time-varying network which is generated by the activity driven model, while the contagion layer is a static network. The activity driven model used for the spreading of information can be depicted as follows:^34^ first, we consider N individuals and assign to each individual i an activity rate $a_{i}=\eta x_{i}$, which is defined as the probability per unit time to create new contacts with other individuals. The activity potentials *x_i_* are assigned according to a probability distribution *F*(*x*) and are bounded in the interval $\xi \leq x_{i}\leq 1$. Then, at each time step, with probability $a_{i}\Delta t$, each individual i is activated and generates *m* connections with other randomly selected individuals. After we build the network, information spreading process, which is the same as in the classic SIS model, will happen on it. At the next time step, we delete all the edges and repeat the above steps. It is obvious that in the model, individuals do not have memory of previous steps. Since many empirical researches show that power-law distribution of *F*(*x*) fits the real world networks,^34^ in the paper, we make *F*(*x*) take the form: $F(x)\propto x^{-\gamma }$. As a result of the coupled dynamical processes, it is worth noting that each individual in this multiplex network can only be in one of the three kinds of states: unaware and susceptible (US), aware and infected (AI), and aware and susceptible (AS). To help readers have a clear understanding of our model, we list the meanings of some key parameters used in our model in Table I.

**FIG. 2.**
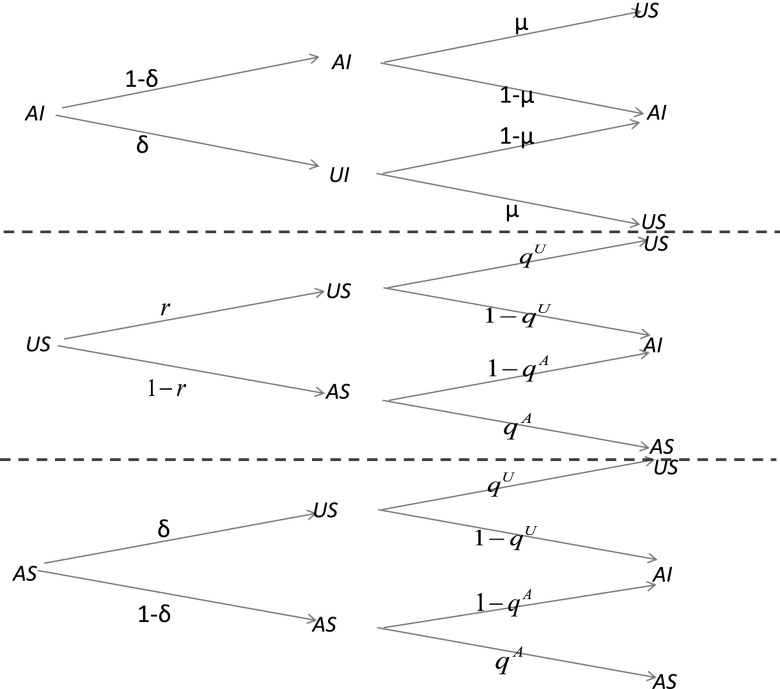
The probability tree for the transitions of states. The states include AI (aware and infected), US (unaware and susceptible), and AS (aware and susceptible). In the probability tree, *μ* represents transition probability from infected to susceptible, *δ* represents transition probability from aware to unaware. Meanwhile, *q^A^* and *q^U^* represent the transition probability for individual not being infected by neighbors if it is aware or unaware, respectively. *r* represents probability for unaware individual staying unaware. The coupled dynamical processes take place consecutive as time goes by.

In the following, with the help of MMCA method which can be illustrated by the way of probability tree,^27–30^ we are able to obtain the epidemic threshold of our model. In Fig. 2, we reveal the possible states and their transitions in our model. Here, let ${\left\{a_{ij}(t)\right\}}_{N\times N}$ represents the adjacent matrix of the awareness layer at time t. Meanwhile, ${\left\{b_{ij}\right\}}_{N\times N}$ is the adjacent matrix of the contagion layer. Since at time t individual i can only be in one of the three states, we denote the probabilities of the three states as $p_{i}^{AI}(t),\;p_{i}^{AS}(t),\;p_{i}^{US}(t)$, respectively. Furthermore, on the awareness layer, we define the probability for unaware individual i staying unaware as $r_{i}(t)$; on the contagion layer, we separately define the probabilities for individual i not being infected by any neighbors as $q_{i}^{A}(t),\;q_{i}^{U}(t)$ if i is aware and unaware.

With respect to the above definitions, considering the coupled dynamical processes we have^27^

$$\begin{array}{c} r_{i}\left(t\right)=\prod_{j=1}^{N}(1-a_{ji}(t)p_{j}^{A}(t)\lambda ),\\ q_{i}^{A}(t)=\prod_{j=1}^{N}(1-b_{ji}p_{j}^{AI}(t)\beta^{A}),\\ q_{i}^{U}(t)=\prod_{j=1}^{N}(1-b_{ji}p_{j}^{AI}(t)\beta^{U}). \end{array}$$ (1)

Hence, according to the probability tree which reveals the transitions of three different states, we can derive a discrete-time version of the evolution of our model by means of Markov chain method, which is

$$\begin{array}{c} p_{i}^{US}(t+1)=p_{i}^{AI}(t)\delta \mu +p_{i}^{US}(t)r_{i}(t)q_{i}^{U}(t)+p_{i}^{AS}\delta q_{i}^{U}(t),\\ p_{i}^{AS}(t+1)=p_{i}^{AI}(t)\mu (1-\delta )+p_{i}^{US}[1-r_{i}(t)]q_{i}^{A}(t)+p_{i}^{AS}(1-\delta )q_{i}^{A}(t),\\ p_{i}^{AI}(t+1)=p_{i}^{AI}(t)(1-\mu )+p_{i}^{US}(t)\{[1-r_{i}(t)][1-q_{i}^{A}(t)]\\ +r_{i}(t)[1-q_{i}^{U}(t)]\}+p_{i}^{AS}(t)\{\delta [1-q_{i}^{U}(t)]\\ +(1-\delta )[1-q_{i}^{A}(t)]\}. \end{array}$$ (2)

For the purpose of calculating the epidemic threshold, it is necessary to explore the stationary solution of the system of Eq. (2). When time t→∞, there exists an epidemic threshold $\beta_{c}^{U}$ for the coupled dynamical processes, which means the epidemic can outbreak only if $\beta \geq \beta_{c}^{U}$. By letting t→∞, the probabilities of three states $p_{i}^{US},\;p_{i}^{AS},\;p_{i}^{AI}$ fulfill the condition that $\underset{t\to \infty }{\lim}p_{i}^{US}(t+1)=p_{i}^{US}(t+1)=p_{i}^{US},\;\underset{t\to \infty }{\lim}p_{i}^{AS}(t+1)=p_{i}^{AS}(t+1)=p_{i}^{AS},\;\underset{t\to \infty }{\lim}p_{i}^{AI}(t+1)=p_{i}^{AI}(t+1)=p_{i}^{AI}$. Since around the epidemic threshold *β_c_*, the infected probability $p_{i}^{AI}=\epsilon_{i}\ll 1$, the probabilities $q_{i}^{A}$ and $q_{i}^{U}$ can be simplified as $q_{i}^{A}\approx (1-\beta^{A}\Sigma_{j}b_{ji}\epsilon_{j}),\;q_{i}^{U}\approx (1-\beta^{U}\Sigma_{j}b_{ji}\epsilon_{j})$, respectively. Therefore, inserting these approximations into Eqs. (2) and omitting higher order items, Eqs. (2) is reduced to the following form:

$$\begin{array}{c} p_{i}^{US}=p_{i}^{US}r_{i}+p_{i}^{AS}\delta ,\\ p_{i}^{AS}=p_{i}^{US}(1-r_{i})+p_{i}^{AS}(1-\delta ),\\ \mu \epsilon_{i}=p_{i}^{US}((1-r_{i})\beta^{A}\Sigma_{j}b_{ji}\epsilon_{j}+r_{i}\beta^{U}\Sigma_{j}b_{ji}\epsilon_{j})\\ +p_{i}^{AS}(\delta \beta^{U}\Sigma_{j}b_{ji}\epsilon_{j}+(1-\delta )\beta^{A}\Sigma_{j}b_{ji}\epsilon_{j}). \end{array}$$ (3)

Afterwards, by analysing the probability $p_{i}^{AI}(\epsilon_{i})$, we can get

$$\mu \epsilon_{i}=(p_{i}^{AS}\beta^{A}+p_{i}^{US}\beta^{U})\Sigma_{j}b_{ji}\epsilon_{j}.$$ (4)

It is clear that $p_{i}^{AI}+p_{i}^{AS}+p_{i}^{US}=1$, where $p_{i}^{A}=p_{i}^{AI}+p_{i}^{AS}$. Noting that $p_{i}^{AI}=\epsilon_{i}\ll 1$, we get $p_{i}^{AI}\approx p_{i}^{A}$ and $p_{i}^{US}=1-p_{i}^{AI}-p_{i}^{AS}=1-p_{i}^{A}$. Hence

$$\mu \epsilon_{i}=\beta^{U}(1-p_{i}^{A})\Sigma_{j}b_{ji}\epsilon_{j},$$ (5)

that is, to say, Eq. (6) can be reduced to

$$\Sigma_{j}[(1-p_{i}^{A})b_{ji}-\frac{\mu }{\beta^{U}}t_{ji}]\epsilon_{j}=0,$$ (6)

where *t_ji_* are the elements of the identify matrix. Let **H** be a matrix whose element *h_ij_* equals $(1-p_{i}^{A})b_{ji}$. Then, it is obvious that the Eq. (6) has nontrivial solutions if and only if $\frac{\mu }{\beta^{U}}$ is the eigenvalue of matrix **H**. Consequently, the epidemic threshold *β_c_* is the one which satisfies $\frac{\mu }{\beta^{U}}=\Lambda_{\text{max}}$, where Λ_max_ is the largest eigenvalue of matrix **H**, and it is easy to get

$$\beta_{c}^{U}=\frac{\mu }{\Lambda_{\text{max}}}.$$ (7)

It is worthy noting that for single layer time varying network, the epidemic threshold can be calculated as^34^

$$\frac{\lambda }{\delta }\geq \frac{1}{m}\frac{1}{〈a〉+〈a^{2}〉},$$ (8)

where $〈a〉=\frac{1}{N}\sum_{j=1}^{N}a_{j},\;〈a^{2}〉=\frac{1}{N}\sum_{j=1}^{N}a_{j}^{2}$. This means that only for $\lambda \geq \frac{\delta }{m(〈a〉+〈a^{2}〉)}=\lambda_{c}$ epidemics can outbreak on top of a single layer time varying network. If we consider the two spreading processes separately, therefore, when $\lambda <\lambda_{c}$, Eq. (7) is reduced to $\beta_{c}=\frac{\mu }{\Lambda_{max}(B)}$ as defined in Ref. 27; thus, $\left(\lambda_{c},\beta_{c}\right)$ also defines a metacritical point for the epidemic spreading in our model.

## IV. SIMULATIONS

### A. SIS model for awareness information diffusion process on time-varying network

**FIG. 3.**
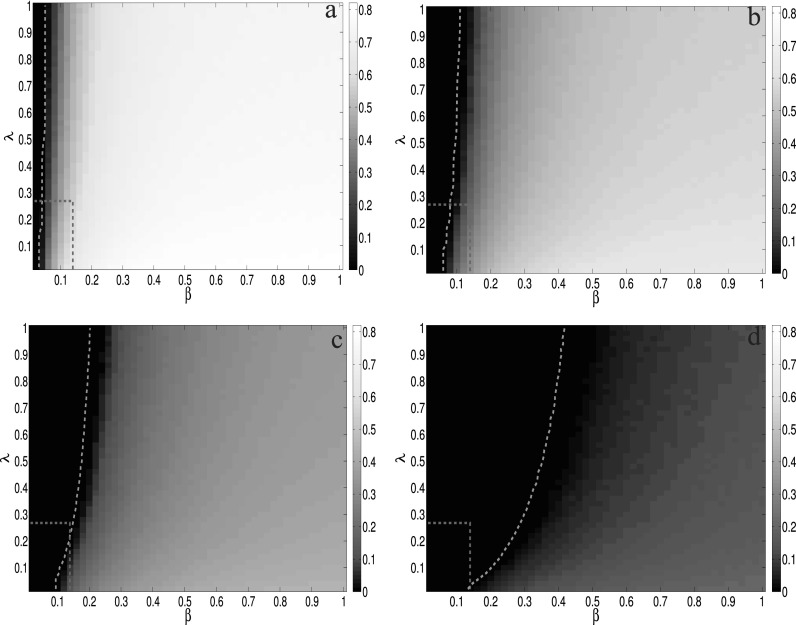
The comparisons of the epidemic threshold between analytic results (MMCA method) and MC simulations as a function of the infected probability *β* and the aware probability *λ*. The green dashed line is the MMCA results for the epidemic threshold and the heatmap represents the density of infected nodes *ρ^I^* obtained by MC simulations under different parameters. From top left to bottom right, the recovery rate *μ* and the forgetting rate *δ* are set to be (a) *μ* = 0.2, *δ* = 0.8, (b) *μ* = 0.4, *δ* = 0.6, (c) *μ* = 0.6, *δ* = 0.4, and (d) *μ* = 0.8, *δ* = 0.2, respectively. The dashed rectangle corresponds to the area where the metacritical points are located, which are bounded by the topological characteristics of each layer $\frac{1}{m(〈a〉+〈a^{2}〉)}$ and $\frac{1}{\Lambda_{\text{max}}(B)}$. The four phase diagrams are obtained by averaging 100 MC simulations for each point in the grid 50 × 50.

Here, to show the validity of the approach discussed above, we have performed extensive MC simulations on multiplex network with 5000 nodes on each layer. The activity driven model, which is used to generate the information spreading layer, is configured as follows: the power law distribution of the activity rate *x_i_* satisfies $F(x)\propto x^{-3}$, and the edge number *m* that one active node can have is 8. Besides, the rescaling factor *η* is set to be 10 and $\xi =10^{-3}$. The other layer is a scale free network, of which the degree distribution $P(k)∼k^{-3}$. After having these settings, it is easy for us to obtain the boundary of the metacritical point $\left(\frac{1}{m(〈a〉+〈a^{2}〉)},\frac{1}{\Lambda_{\text{max}}(B)}\right)$, which equals (0.2677, 0.1390) in our case. In Fig. 3, we show the comparison between analytic results calculated by Eq. (7) and MC simulations under various conditions. All the simulations start from a fraction *ρ*_0_ of randomly chosen infected nodes and *ρ*_0_ is fixed to be 0.2. At each time step, all the neighbours of an infected node become infected with the same probability *β* and the infected node recovers at a rate *μ*. The same process fits for the spreading of information—what we need to do is just replace the infected probability *β* and recovery rate *μ* with the aware probability *λ* and forgetting rate *δ*, respectively. Iterate the rules of the coupled dynamical processes with parallel updating until the density of infected nodes *ρ^I^* is steady. In order to reduce the fluctuation of the density *ρ^I^*, we make time average that satisfies $\rho^{I}=\frac{1}{T}\Sigma_{t=t_{0}}^{t=t_{0}+T-1}\rho^{I}(t)$ and take T = 100 ($t_{0}=901$).

From the results above, it is clear that the MMCA method has a high accuracy to predict the epidemic threshold, no matter what values other parameters are set to be. Besides, there are also some interesting phenomena revealed by our model. When the value of *μ* is large, while the value of *δ* is small, the value of aware probability *λ* has an obvious effect on the epidemic threshold. The reason is that at this condition, it is easy for the aware nodes to stay in the aware state, while at the same time, the recovery ability of the nodes is strong. These two effects lead the epidemics to be difficult to outbreak. Hence, if we increase *λ*, which means that the unaware nodes can become aware more easily, the epidemic threshold becomes larger. However, if *μ* is small and *δ* is large, it is easy for the epidemics to outbreak. And then, even if we increase *λ* and nodes become aware with higher probability, the large value of *δ* makes nodes forget the epidemics in a short time, which will decrease the spread speed of awareness. That is why in this case, *λ* has a little effect on the epidemic threshold. Furthermore, note that there also exists a region bounded by $\left[\frac{1}{m(〈a〉+〈a^{2}〉)},\frac{1}{\Lambda_{max}(B)}\right]$ where the metacritical point is localized, as illustrated in Ref. 27.

**FIG. 4.**
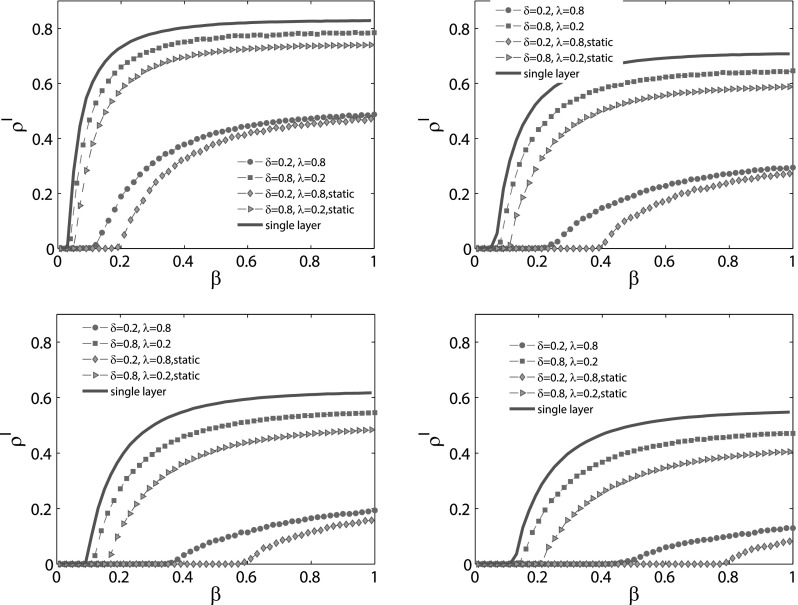
MC simulations for epidemic spreading on multiplex network (red lines) and single layer network (blue lines), as well as a two-layer static multiplex network (green lines). The topology structure of the single layer network is the same as the contagion layer of the multiplex network defined above. As for the static network, except that the awareness layer is a static network, its topology structure is totally the same as that of the time-varying network. On each panel, according to different values of the aware probability *λ* and the forgetting rate *δ*, we plot four dashed lines for the coupled dynamical processes. The red circle and green diamond line are under the condition when *δ* = 0.2 and *λ* = 0.8, while for the red square and green triangle line, *δ* = 0.8 and *λ* = 0.2. As for the recovery rate *μ*, the value is set to be (a) *μ* = 0.2, (b) *μ* = 0.4, (c) *μ* = 0.6, and (d) *μ* = 0.8. Each line is obtained by averaging 50 independent MC simulations.

In order to explore the difference of epidemic spreading between our model with other models, including the classic SIS model on a single layer network and a static multiplex network, in Fig. 4, we compare these models under different initial conditions. The multiplex network, of which the topology structure of epidemic spreading layer is the same as that of the single layer network, is the one defined in Fig. 3. As shown in Fig. 4, with the help of awareness spreading, not only the epidemic threshold, but also the final density of the infected nodes *ρ^I^* is smaller than that of the single layer. However, compared with the static multiplex network, it is obvious that the time variability of the awareness layer weakens the suppression effects on the spreading of epidemics, since the epidemic threshold is smaller and the final epidemic size is larger than that of the static multiplex network.

### B. Threshold model for awareness information diffusion process on time-varying network

**FIG. 5.**
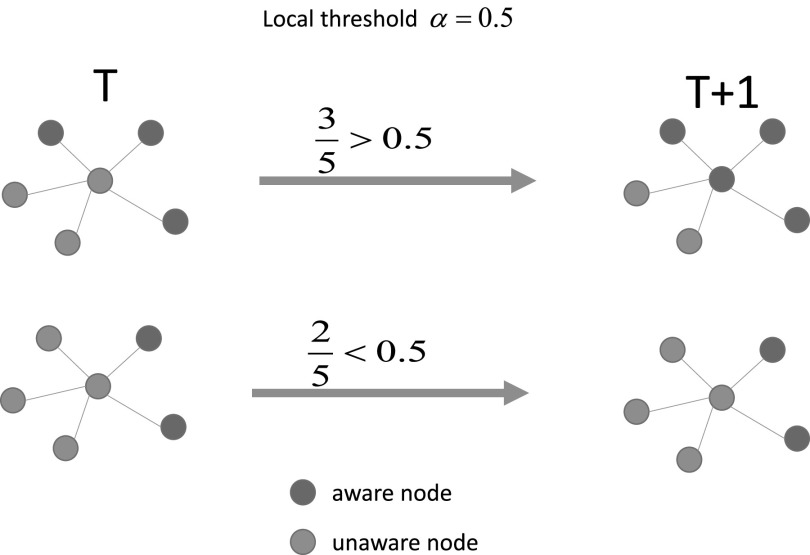
Illustration of the threshold model used to simulate the spreading of information. Only if the ratio between the number of aware neighbors and its degree larger than the threshold value *α*, the unaware individual can become aware. Here, in the toy model, the threshold is set to be 0.5. The red individuals are aware, while the blue ones are unaware.

As for the information spreading, apart from the topology structure of network, the way information spreads is also very important. Since in reality, individuals exhibit herd-like behavior because they are making decisions based on the actions of other individuals, which is called information cascade,^39^ instead of the classic SIS model, we use a threshold model to simulate the spreading of information. In the threshold model, for an unaware individual, awareness can come from two sources: the ratio between the number of aware neighbors and all its connections which is also known as node degree surpasses the critical value (local threshold *α*) or it is already infected. In Fig. 5, we illustrate the threshold model defined on the time varying layer.

**FIG. 6.**
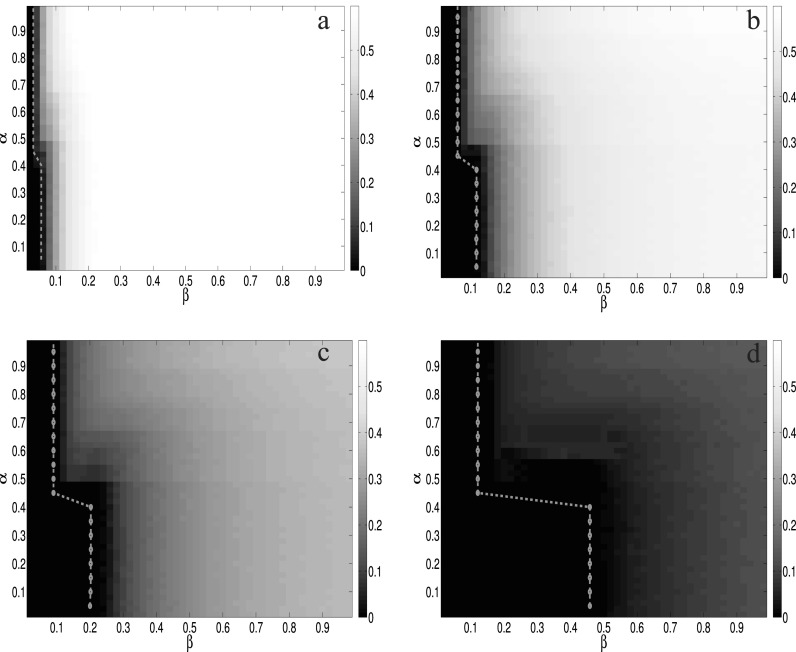
The comparisons of the epidemic threshold between numerical results (MMCA method) and MC simulations as a function of the infected probability *β* and the local threshold *α*. The green dotted line corresponds to the epidemic thresholds calculated by the MMCA method, while the heatmap represents the density of the infected nodes *ρ^I^* obtained by MC simulations under different parameters. From top left to bottom right, the recovery rate *μ* and the forgetting rate *δ* are set to be (a) *μ* = 0.2, *δ* = 0.8, (b) *μ* = 0.4, *δ* = 0.6, (c) *μ* = 0.6, *δ* = 0.4, and (d) *μ* = 0.8, *δ* = 0.2, respectively. The four phase diagrams are obtained by averaging 100 MC simulations for each point in the grid 50 × 50.

Therefore, the multiplex network is made up of two layers with different models on each layer, as described in Ref. 28. For the purpose of calculating the epidemic threshold, we need to change the expression of function *r_i_* as follows:

$$r_{i}\left(t\right)=H(\alpha -\frac{\sum_{j}a_{ji}(t)p_{j}^{A}(t)}{k_{i}(t)}),$$ (9)

where $a_{ji}(t)=1$ if there is a connection between node i and node j at time t, otherwise $a_{ji}(t)=0$. Besides, $k_{i}(t)$ is the degree, the number of its connections, of node i at time t. **H**(x) is a Heaviside step function, i.e., if *x* > 0, **H**(x) = 1, else **H**(x) = 0. Note that since function $q_{i}^{A}(t)$ and $q_{i}^{U}(t)$ (Eq. (1)) represent the transformation between state S and state I, there is no need to change these two functions. Then after the same derivation, we can also get the numerical result of the epidemic threshold

$$\beta_{c}=\frac{\mu }{\Lambda_{\text{max}}},$$ (10)

where Λ_max_ is the same as before, i.e., the largest eigenvalue of the matrix **H** defined following Eq. (6). In Fig. 6, we crosscheck the numerical results with extensive MC simulations on top of the same multiplex network defined above. The results show that the agreement between MMCA method and MC simulations is quite well. Besides, as illustrated in Ref. 28, the two stage effects, which means that there exists a sharp like transition for the epidemic threshold at α≈0.5, also exists in our model irrespective of the value of *δ* and *μ*. Since in our model, the information spreading layer is a time varying network, which is very different from the scale-free network or Erdős-Rényi network, the existence of the two stage effects verifies again that the phenomenon does not rely on the structure of the network, but is a result of the coupled dynamical processes itself. In addition, there is another interesting phenomenon that in Figs. 3 and 6, the agreement between the MMCA approximation and the MC simulations seems to degrade as *μ* increases and *δ* decreases. Actually, a large value of *μ* and a small value of *δ* can lower the final epidemic size and heighten the epidemic threshold. If we focus on the absolute discrepancy between the MMCA method and MC simulations, it seems that the discrepancy is large on the occasion. However, from a macro point of view, for example, the error rate which equals $\frac{\beta_{MC}-\beta_{MMCA}}{\beta_{MC}}$, we find the MMCA method always has a good performance whatever *μ* and *δ* is since the error rate locates in the range of 0%–5%.

**FIG. 7.**
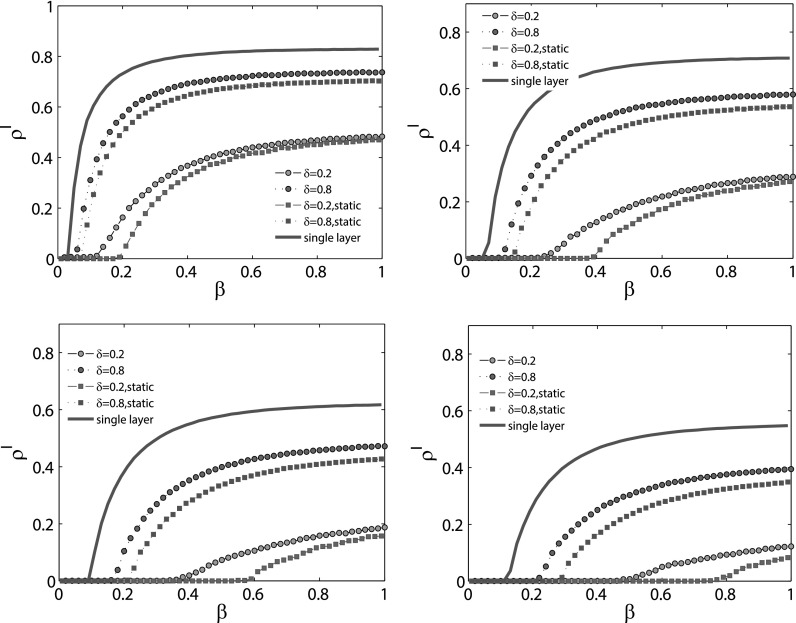
MC simulations for epidemic spreading on multiplex network (circle lines) and single layer network (blue lines), as well as a two-layer static multiplex network (square lines). The topology structure of the single layer network is the same as the contagion layer of the multiplex network defined above. As for the static network, except that the awareness layer is a static network, its topology structure is totally the same as the time-varying network. The recovery rate *μ* is set as follows: (a) μ=0.2, (b) μ=0.4, (c) μ=0.6, and (d) μ=0.8. In each panel, the dashed lines represent the condition when the forgetting rate δ=0.2 and for the dotted lines, δ=0.8. Besides, the awareness probability is set to be 0.2. Every line is obtained by averaging 100 MC simulations.

Moreover, we also compare our model on the multiplex network with other models, including the SIS model on the single layer network and the threshold model on the static multiplex network, as can be seen in Fig. 7. Obviously, as a result of information spreading, the epidemic threshold of our model is larger than that of the single layer case. The final epidemic size is also the smaller one, which means that information spreading can always suppress the spreading of epidemics. The differences of the spreading process between the static multiplex network and the time varying case also crosscheck the findings about time variability of the awareness layer topology, as discussed in Fig. 4.

**FIG. 8.**
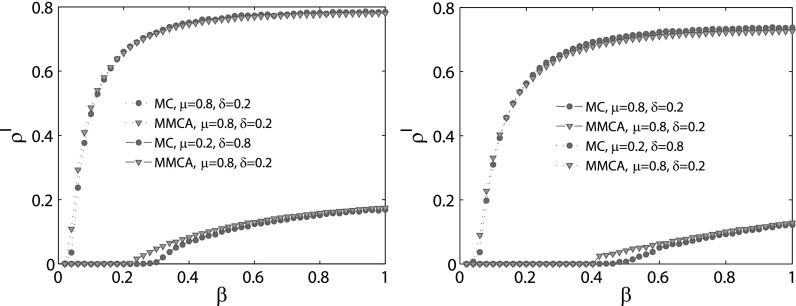
The comparison of the steady density of the infected nodes $\rho_{i}^{I}$ between MMCA method (triangles) and MC simulations (circles). Left panel is obtained under the SIS model, while right panel is obtained under the threshold model. The other parameters are set as follows: λ=0.2,α=0.2. Each line of the MC simulations is obtained by averaging 100 independent experiments.

From the results above, we can find that the epidemic threshold calculated by the MMCA method has a good agreement with MC simulations. Since according to Eqs. (1)–(3), it is easy for us to obtain the steady density of the infected nodes *ρ^I^*, here in order to have a better understanding of the MMCA method, we plot *ρ^I^* as a function of *β* by means of the MMCA method and MC simulations, as illustrated in Fig. 8. The findings reveal that the agreement between the MMCA method and MC simulations decreases with the increase in *μ* and the decrease in *δ*, which is the same as in Figs. 3 and 6.

**FIG. 9.**
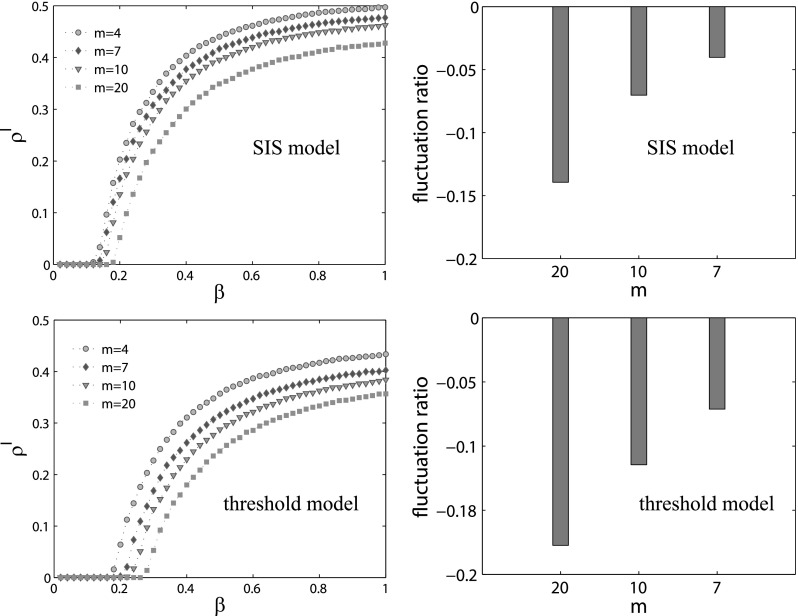
The effects of different time varying topologies on the spreading of epidemics under the SIS model and the threshold model. For each model, we plot four dotted lines through changing the parameter m, namely, 4, 7, 10, and 20, which can produce different time varying networks. The left two panels show the percent of the infected nodes *ρ^I^* as a function of *β*. Meanwhile, the right panels correspond to the fluctuation ratio calculated by Eq. (11).

Since we propose two models to explore the coupled dynamical processes on multiplex network with awareness layer being a time varying network, it is of most interest for us to explore the effects that the varying topology has on different models. As for the activity driven model, m is a critical parameter for the topology structure of the resulting time varying network. Accordingly in the following, we perform many simulations on the two models by setting m to be 4, 7, 10, and 20. Furthermore, in order to study to what extent the coupled dynamical processes is affected by varying topology, a new index named fluctuation ratio is introduced, which is defined as follows:

$$FR(t)=\frac{f^{\left(t\right)}-f^{\left(4\right)}}{f^{\left(4\right)}},$$ (11)

where $f^{\left(t\right)}$ is the final epidemic size of the coupled dynamical processes when m is set to be t. It is clear that for both models, with the increase in m, the epidemic threshold and final epidemic size become smaller. The reason is that m represents the number of edges each active node can connect, the larger the m is, the denser is the network. As a result, the spreading process on the dense network is quicker, which makes the nodes become aware easier. This will then suppress the spreading of epidemics. Moreover, the results also show that the threshold model is more susceptible to random changes in the topology of time varying network, since the fluctuation of the final epidemic size, as well as the epidemic threshold, is much larger in this case (Fig. 9).

**FIG. 10.**
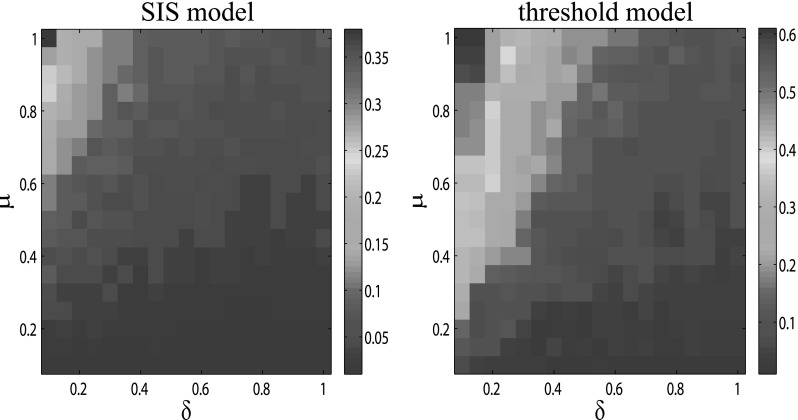
The phase space of *μ* and *δ* with color corresponding to the effect of *λ* (SIS model, left panel) or *α* (threshold model, right panel). The settings of the multiplex network are the same as those described in Fig. 3. Importantly, each panel is obtained by two steps: for each pair values of (*μ*, *δ*), first, we calculate the epidemic thresholds by letting *λ* (*α*) be 0.8 and 0.2, respectively. Then, the final value, which is shown on each panel, equals the epidemic threshold at *λ* = 0.8 (*α* = 0.2) minus the epidemic threshold at *λ* = 0.2 (*α* = 0.2).

In addition, as described above, the change of the varying topology of awareness layer can result in different spreading processes for awareness, and then, the epidemic spreading process is also changed. It is also meaningful for us to quantify to what extent the epidemic spreading is affected by the awareness parameter, which is *λ* for the SIS model or *α* for the threshold model. Thus, in the following, with the help of MMCA method, we study the evolution of epidemic threshold according to the values of *μ* and *δ*. In order to have a better understanding of the effect of *λ* or *α*, we plot the phase space of *μ* and *δ*, and the color of it corresponds to the value which is the epidemic threshold at *λ* = 0.8 (*α* = 0.2) minus the epidemic threshold at *λ* = 0.2 (*α* = 0.8), as illustrated in Fig. 10. The results show that the awareness parameter has a more obvious effect on the threshold model since the value of $\beta_{\alpha =0.2}-\beta_{\alpha =0.8}$ is much larger than that of the SIS model, especially when *δ* is small and *μ* is large. Taking the large fluctuation rate phenomenon studied above into account, we find that the threshold model is “frail” under the configuration of our multiplex network.

## V. CONCLUSION

As a summary, in this paper, through considering the interactions among individuals as a time varying network where the awareness diffusion process occurs, we have explored the effects that the spread of awareness has on epidemic spreading. With the help of the multiplex network, we are able to model the interplay between these two kinds of dynamical processes. Afterwards, we build the probability tree for the coupled dynamical processes to reveal the transitions among different states of individuals. Using the MMCA method, the epidemic threshold can be obtained by solving an eigenvalue problem. Regardless of whether the information spreading process is defined as the SIS model or the threshold model, our numerical results of epidemic threshold show high accuracy compared with extensive MC simulations. Specifically, when the information spreading process is the SIS model, the coupled dynamical processes make it difficult for epidemics to outbreak and can also lower the density of the infected individuals. However, if the information spreading process is assumed to be a threshold model, the epidemic threshold exists as a sharp like transition when the local threshold α≈0.5 though all the epidemic thresholds are smaller than the single layer threshold.

Furthermore, compared with the static multiplex network with the same dynamics, we find that the temporal setting hinders the spreading of awareness which directly affect the epidemic threshold. These results show the importance of taking the information spreading process into account when we try to control the spread of epidemics. From the point of view of information diffusion model, our results show that temporal changes in the topology also hinder the spread of awareness, especially for the threshold model. Since in reality with the burst development of social networks, the way how information spreads is a very complex problem for scientists from many different fields, proposing a proper model to study it is of great significance. Although in this paper, we use the activity driven model to construct the propagation path of information, there are still many other different approaches to realize the goal, for example, bursty model^36^ and so on. Only if we have a good understanding of the two dynamical processes, which include information spreading and epidemic spreading, it can be possible for us to explore the coupled effects between them. Besides, the results show that changes in the topology of the awareness layer could change the location of the meta-critical point. This is particularly important since a slight delay or advance in the epidemic could have important implications to vaccination strategies.^40^ Overall, our results give us some useful suggestions about how to model this kind of coupled system and also shed light on new strategies on restraining epidemics.

## Figures and Tables

**TABLE I.** The details of some key parameters.

Parameter	Description
*λ*	Transition probability from unaware to aware
*δ*	Transition probability from aware to unaware
*β^U^*	Infection rate for unaware individual
*β^A^*	Infection rate for aware individual
*μ*	Recovery rate for infected individual
*x_i_*	The activity potential of individual i
*a_i_*	The activity rate of individual i
*η*	Rescaling factor of the activity potential
*ξ*	The lower bounder of individual's activity potential
*m*	The number of links an active individual generates
*α*	The local threshold ratio
